# Risk Assessment of Mycotoxins in Stored Maize Grains Consumed by Infants and Young Children in Nigeria

**DOI:** 10.3390/children4070058

**Published:** 2017-07-10

**Authors:** Modupeade C. Adetunji, Olusegun O. Atanda, Chibundu N. Ezekiel

**Affiliations:** 1 Department of Biological Sciences, McPherson University, Seriki Sotayo 110117, Ogun State, Nigeria; ogunrinumodupe@gmail.com; 2 Department of Microbiology, Babcock University, Ilishan Remo 121103, Ogun State, Nigeria; chaugez@gmail.com

**Keywords:** exposure assessment, children, infants, maize, risk assessment

## Abstract

Maize is a major complimentary food for infants (0–4 years) and young children (5–12 years) in Nigeria. In this study, we assessed the risk of exposure of infants and young children (IYC) to some major mycotoxins in stored maize grains from five agro-ecological zones of Nigeria. The probable daily intake approach was employed to determine exposure to five mycotoxins while the margin of exposure (MOE) and population at risk of primary hepatocellular carcinoma approaches were used to characterize the risk of consuming aflatoxin contaminated maize. Infants and young children in the Derived Savannah zone are more exposed to aflatoxins, ochratoxins, and zearalenone while those in the Northern Guinea Savanna zone are mainly exposed to deoxynivalenol and fumonisins. The mean national MOE for infants and children were 0.12 and 0.3 respectively while the risk of developing primary liver cancer was estimated at 152.7 and 61.1 cancer/year/100,000 population of infants and children, respectively. Infants and young children consuming mycotoxin contaminated maize in Nigeria are therefore vulnerable to the adverse health effects. Mycotoxin contamination of maize is still a challenge in Nigeria; mitigation efforts should target the value chain and stricter tolerable limits should be enforced.

## 1. Introduction

Maize (*Zea mays* L.) is one of the most important agricultural commodities in the world and is the third most traded cereal after wheat and rice with a production of 745 billion kg over 1.60 million m^2^ hectares of land in 2012, and Nigeria was ranked the world’s eighth largest producer of maize by the Food and Agricultural Organization of the United Nations (FAO) in 2013 [1]. Maize is one of the cereals prepared by mothers separately or in combination with other cereals (e.g., sorghum, wheat, or millet) as weaning foods for infants less than five years old [2,3,4]. A major challenge limiting the food safety and nutritional benefits of maize in infants and young children (IYC) is contamination of maize grains by mycotoxins.

Frequent contamination of maize grains by different fungi are known to cause major health problems in humans. Aflatoxins are known to be carcinogenic, mutagenic, nephrotoxic, immune suppressant, and could lead to stunting growth in children [5]. Colonization of maize grains with *Fusarium* spp. leads to the production of fumonisins (FB_1_, FB_2_ and FB_3_). FB_1_ has been associated with kidney damage, liver damage, as an agent of leukoencephalomalacia and as a possible human carcinogen (Group 2B) [5,6]. Infants and children below 12 years are more vulnerable to the effects of mycotoxins because of their less developed immune systems and high intake of foods and water per kg body weight [7,8]. In addition, the co-occurrence of mycotoxins is an increasing concern due to the possible combined exposure risks posed to humans which may be observed as increased toxicity and carcinogenicity; especially compared to effects from single mycotoxins [7]. The Joint FAO/World Health Organization (WHO) Expert Committee on Food Additives (JECFA) at different times established Provisional Maximum Tolerable Daily Intakes (PMTDI) for some mycotoxins to protect consumers’ health [2,4].

Periodic exposure assessments to different mycotoxins in foods from different regions of the world have been documented [2,4,9,10,11]. Previous studies had also reported the incidence and co-occurrence of mycotoxins in cereals. Kimanya et al. [4] reported the occurrence of high levels of aflatoxins (158 μg·kg^−1^) and total fumonisin (11,048 μg·kg^−1^) in Tanzanian maize. Similarly, Adetunji et al. [3] reported high levels and co-occurrence of aflatoxin B_1_ (AFB_1_; max.: 6738 μg·kg^−1^), fumonisin B_1_ (FB_1_; max.: 10,447 μg·kg^−1^), and zearalenone (ZEN; max.: 2044 μg·kg^−1^) contamination in 67, 93, and 17%, of stored maize grains in Nigeria. Furthermore, aflatoxins (588 μg·kg^−1^), citrinin (16,773 μg·kg^−1^), fumonisins (2294 μg·kg^−1^) and ZEN (205 μg·kg^−1^) were reported in a batch of yellow maize used for the production of *ogi* (fermented maize gruel) [12] while FB_1_ was reported to also occur in maize and *ogi* from Nigeria at maximum concentrations of 8222 μg·kg^−1^ and 1903 μg·kg^−1^, respectively [13].

Despite the available data on mycotoxin occurrence in maize in Nigeria and the wide usage of maize in IYC nutrition in the country, literature is lacking information on exposure and risk assessment of mycotoxins found in maize and maize-based products intended for IYC nutrition. Recently, there was a report on exposure assessment of Nigerian adults to mycotoxins through consumption of maize grains [9]. In order to complement the report, we determined the exposure levels of Nigerian infants and children aged 0–4 years and 5–12 years, respectively, to mycotoxins through maize consumption and assessed the associated risks as a result of its consumption since maize is a staple grain used in IYC nutrition in the country.

## 2. Materials and Methods 

### 2.1. Survey Sites

Surveys were conducted between August 2011 and February 2012 in five agro-ecological zones (AEZs) of Nigeria where maize is predominantly produced [3]. The AEZs and their states were: Derived Savanna (DS: Ekiti, Nasarawa, Ondo, Osun and Oyo states), Humid Forest (HF: Lagos and Ogun states), Northern Guinea Savanna (NGS: Kaduna state), Southern Guinea Savanna (SGS: Niger state), and Sudan Savanna (SS: Kano and Sokoto states).

#### Maize Samples

Sampling of maize and sample preparation prior to mycotoxin analysis were carried out as described by Adetunji et al. [3]. A total of 70 composite samples (3 kg each) were collected across the five AEZs (DS (*n* = 33), HF (*n* = 4), NGS (*n* = 11), SGS (*n* = 11) and SS (*n* = 11)) and analyzed for aflatoxins, fumonisins, deoxynivalenol, ochratoxins, and zearalenone [3]. 

### 2.2. Exposure Assessment of Maize Consumers to Mycotoxins

The concentrations of mycotoxins as previously reported by Adetunji et al. [9] were employed in the assessment of exposure by IYC in this study. The mycotoxins included: total aflatoxin (sum of AFB_1_, AFB_2_, AFG_1_, and AFG_2_), AFB_1_, deoxynivalenol (DON), total fumonisin (sum of FB_1_, FB_2_, and FB_3_), ochratoxin A (OTA), and ZEN. The method of Liu and Wu [14] was adopted to estimate IYC exposure levels to mycotoxins found to be predominant in the grains. The average maize consumed (57 g/person/day) in Nigeria was adapted from the WHO Global Environment Monitoring System (GEMS)/food consumption cluster diets database as there is no data for average maize consumption for IYC in Nigeria [15]. The average maize consumed was multiplied by the mean of means of aflatoxin concentration of the stored grains in the various AEZs, and the product divided with the average body weight of 10, 25 [2], and 60 kg [14] for infants (0–4 years), children (5–12 years) and adults (18–65 years), respectively, as shown below. 

Exposure assessment data on adults were obtained from a previous report by the author to enable comparison with exposure data for IYC [9].

$${\mathrm{PDI}}_{m}=\left(\mu_{\bar{X}}{\;\times \;C}_{c}\right)/B_{w\;}$$

where

${\mathrm{PDI}}_{m}$: Probable daily intake for each mycotoxins (μg·kg^−1^ bw·day^−1^)
$\mu_{\bar{X}}$C: Mean of means of mycotoxin concentration/AEZ
$C_{c}$*:* Average consumption of maize in Nigeria
$B_{w\;}$: Body weight for the population groups (infants, children, and adults)

### 2.3. Risk Characterisation for Mycotoxins among Maize Consumers

#### 2.3.1. Risk Characterization for Aflatoxins

Risk characterization for genotoxic and carcinogenic compounds such as aflatoxins should be based on Margin of Exposures (MOEs), which was calculated by dividing the Benchmark dose lower limit (BMDL) for aflatoxins 170 ng·kg^−1^ bw·day^−1^ [16] by the toxin exposure for each infant or young child. Data was calculated for adults for comparison with those of IYC. In cases where MOEs were lower than 10,000, a public health concern is indicated which implied that aflatoxin exposure above 0.017 ng·kg^−1^ bw·day^−1^ (as obtained by dividing 170 ng·kg^−1^ bw·day^−1^ by 10,000) represented a risk of public health concern.

#### 2.3.2. Estimated Liver Cancer Risk due to Consumption of Maize Grains

The risk of developing liver cancer among Nigerian IYC due to the consumption of the contaminated grains was estimated for AFB_1_ only, because the ingestion of the toxin can be linked to the development of liver cancer [16]. This involved estimating the population cancer risk per 100,000 which was obtained by multiplying the PDI_m_ value with the average hepatocellular carcinoma (HCC) potency figure from individual potencies of HBsAg-positive and HBsAg-negative groups. The HBsAg-positive prevalence rate was assumed to be 25% for developing countries and 75% (100–25%) was extrapolated for HBsAg-negative groups [17]. Cancer risk data for adults were also calculated and compared with those of IYC.

Population risk = Exposure × Average potency

Average potency = (0.3 × 0.25) + (0.01 × 0.75) = 0.0825 cancers per year per 100,000 per ng AFB_1_ kg^−1^ bw day^−1^

#### 2.3.3. Risk Characterization for Non-Genotoxic and Non-Carcinogenic Mycotoxins

The risk characterization for non-genotoxic and non-carcinogenic mycotoxins (%Tolerable Daily Intake (TDI)) was determined by dividing the PDI_m_ with the TDI_m_ and multiplying the product with 100 as shown in the equation below. TDI data for adults was also calculated to enable comparison with data for IYC.

%TDI = (PDI_m_/TDI_m_) × 100 (1)

## 3. Results

### 3.1. Exposure and Risk Assessment

Table 1 shows the exposure and risk assessment data for aflatoxins among the different population groups that consume maize in Nigeria. The results showed high exposure levels to aflatoxins among infants and children with a mean PDI (national) of 1909.1 and 763.6 ng·kg^−1^ bw·day^−1^ respectively, as compared to the adult groups with a mean national PDI of 318.2 ng·kg^−1^ bw·day^−1^. The respective mean and maximum total aflatoxin exposure levels for infants (3402.1 and 42,066 ng·kg^−1^ bw·day^−1^) and children (1360.8 and 16,826.4 ng·kg^−1^ bw·day^−1^) within the DS zone were the highest across the AEZs whilst infants (782.8 and 3021 ng·kg^−1^ bw·day^−1^) and children (313.1 and 1208.4 ng·kg^−1^ bw·day^−1^) in the SS zone had the least exposure. The mean exposure levels of all categories of maize consumers to aflatoxins across the AEZs exceeded the 0.017 ng·kg^−1^ bw·day^−1^ permissible exposure threshold by more than 45,000fold. Consequently, the mean MOEs for the population categories were 0.12, 0.3, and 0.70 for infants, children, and adults respectively across the AEZs which were far less than the 10,000 permissible limit, thus indicating a public health risk.

The estimated population of infants and children at risk of primary liver cancer is also indicated in Table 1. With a mean national dietary AFB_1_ exposure estimated at 1850.7 and 740.3 ng·kg^−1^ bw·day^−1^ for infants and children, respectively, about 152.7 and 61.1 Nigerian infants and children per 100,000 population are at risk of primary liver cancer as against 25.5 adults previously reported [9]. Infants (240 out of every 100,000 population) in the DS are more at risk of HCC than infants and children in other AEZs.

### 3.2. Risk Characterization of Non-Genotoxic and Non-Carcinogenic Mycotoxins Based on Probable Daily Intake

Table 2 reveals the risk of exposure of IYC to other non-genotoxic and non-carcinogenic mycotoxins such as fumonisins, DON, OTA, and ZEN with their corresponding %TDIs. The risk of exposure of infants in the NGS zone of the country to fumonisin was the highest (%TDI: 849.2) compared to the risk in other zones SGS, HF, DS, and SS (%TDI: 762.4, 688, 613.8, and 197.9, respectively). These values are several-hundred-fold higher than the 2 μg·kg^−1^ bw provisional maximum TDI (PMTDI) established for fumonisins by JECFA [18,19,20].

Infants and children in the DS zone were exposed to 732.1 and 292.8 μg·kg^−1^ bw·day^−1^ of OTA through maize consumption in their diets with %TDI of 4306.6 and 1722.6, respectively, which is more than 200 times the regulated MTL value of 17 ng·kg^−1^ bw·day^−1^. Infants and children at the NGS zone had high risks of exposure to DON with TDI values which were 63 and 25 times higher than the recommended tolerable daily intake of 1 μg·kg^−1^ bw·day^−1^. Infants, children, and adult consumers in the DS zone had the lowest %TDI of 29.2, 11.7, and 4.9 respectively for DON, and were followed by %TDI of 29.4, 11.8, and 5.0, respectively for the SGS zone. Based on this study, consumers in the SS and HF zones of the country had no risk of exposure to ZEN due to no contamination of their maize grains, while consumers in the NGS zone had the least %TDI of 2.1, 0.8, and 0.3 for infants, children, and adults respectively. Consumers in the DS zone were at a high risk of exposure to ZEN with %TDI of 395.6, 158.3, and 66 which were 1582, 633, and 264 times higher than the tolerable daily intake of 0.25 μg·kg^−1^ bw·day^−1^.

## 4. Discussion

In this study, we employed the PDI approach to determine exposure to aflatoxins and four other major mycotoxins, while the MOE and population at risk of primary hepatocellular carcinoma approaches were used to characterize the risk of consuming maize contaminated with aflatoxins. The mean MOE data across the population showed a trend of infants > children > adults. The aflatoxin exposure values obtained for infants and children in this study were about 16- and 6-folds higher than the values obtained for 13 children (786 ng·kg^−1^ bw·day^−1^) who consumed maize-based complementary foods in Rombo, Tanzania [4]. The high PDI and MOE values obtained in our study for maize consumers indicate the extent of risk of aflatoxicosis these consumers may be exposed to. The risks and effects of aflatoxin exposures across populations are usually more pronounced in IYC and can be mostly expressed as stunting, immune suppression, impaired growth, and cognitive functions [6,22,23].

Daily consumption of low concentrations (below regulatory limits) of aflatoxins especially AFB_1_ for a considerable length of time may culminate in the development of hepatocellular carconima (HCC). The current burden of HCC attributable to aflatoxin exposure in developing countries including Nigeria is less estimated [24], thus there is need for increased exposure and risk assessment studies in these regions and among vulnerable populations such as IYC. Since the desired level of protection is that dietary aflatoxin exposure should not cause an increase of 1/100,000 cases in the relevant population over a life time, then allowable aflatoxins in maize and peanuts should be ‘As Low As Reasonably Achievable’ (ALARA). It was predicted that at most 9 ng·g^−1^ aflatoxin in foodstuff would cause an increase of 1 HCC case per 10,000 population [25]. Nigeria is one of the countries with high HBsAg-positive prevalence rate of 13.2% [23]; therefore, efforts towards aflatoxin mitigation in the maize value chain should be coordinated and well-targeted. Stricter tolerable limits should also be enforced in order to provide safe food for the population especially for IYC and thus lower the risk of aflatoxin-mediated HCC.

The mean national PDI for total fumonisin and %TDI obtained in this report were much higher than the report of exposure in rural Tanzania where the risk of fumonisin exposure in 26 infants exceeded the PMTDI limit of 2 μg·kg^−1^ bw based on the fumonisin concentration of 11,048 μg·kg^−1^ in the maize grains [26]. Shephard also reported a fumonisin concentration of 10,140 μg·kg^−1^ in South African maize [27]. This is probably due to the high daily consumption rate of 397 g·day^−1^ and 356 g·day^−1^ in South Africa and Tanzania, respectively. Consumption of food contaminated with high concentration of fumonisin greater than the PMTDI has been associated with shorter height (1.3 cm) and lighter weight (328 g) in children [28].

Ochratoxins are notable nephrotoxic compounds that could pose a risk to consumers of ochratoxin contaminated products. There was no record of OTA contamination or exposure in the NGS and HF zone. This is in contrast to the report of Sibanda who detected a high level (150 μg·kg^−1^) of the toxin in maize from northern Nigeria comprising of NGS zone [29]. The respective mean national PDI and %TDI for infants (193.1 and 2061.7) and children (140.0 and 824.7) in the country showed that IYC in Nigeria are at high risk of OTA exposure from maize grains. In addition to its nephrotoxicity, OTA has been experimentally shown to be teratogenic and immunosuppressive. The IARC classified ochratoxin as a possible human carcinogen [30]. Furthermore, as an enzyme inhibitor, it affects lipid per oxidation and has been implicated in Balkan Endemic Nephropathy (BEN) in humans [31]. The contamination level of DON in Nigeria maize is however lower than the range of 57–825 μg·kg^−1^ reported by Kimanya in various mixed maize meals fed to children in Tanzania [4].

A vast number of researchers have reported that the risk of mycotoxins exposure in Africa is higher than what is obtainable in other parts of the world and this is as a result of the high rate of consumption of maize-based foods, especially by infants as weaning or complimentary foods [4,26,32]. The average maize consumption rate of 57 g/person/day from GEMS/Food consumption cluster diet data base [21] used for Nigeria is far lower than the 100 g/child/day reported by Kimanya et al. [4] for Tanzanian children but higher than that recommended by GEMS/Food Regional Diets of 8.8 g·day^−1^ for Europe, 31.2 g·day^−1^ for East Asia, and 40 g·day^−1^ for Latin America [21]. There is currently no set standard value for average daily consumption for maize in Nigeria hence the GEM standard value of 57 g·day^−1^ was adopted in this study. In addition, Shephard et al. [27] and Kimanya et al. [4] had shown that maize consumption can vary by as much as 100-fold between developed countries and under developed countries, particularly in Africa.

## 5. Conclusions

We report, for the first time, mycotoxin risk assessment for IYC from consumption of a major staple (maize) in Nigeria and—more importantly—the risk of liver cancer in this highly susceptible population. Infants and young children in Nigeria are at high risk of exposure to the five major mycotoxins reported in this study through consumption of maize as complementary foods. Adults are less susceptible to the risk of exposure to toxins due to the weight differences between them and the IYC populations, although the health effects due to mycotoxin exposure affect all population categories. Therefore, a major limitation of this research is the assumption of the same consumption rate for both adults and the IYC population. Nigeria is yet to have a database for daily consumption rate for maize and some other cereals for different age groups in the country. Since the risk of exposure decreases with increases in body weight, our study has likely underestimated the mycotoxin risk in the IYC populations. Hence, exclusive breastfeeding should be encouraged in the country, especially in the first six months of life and complementary food components should be diverse to include other cereals such as sorghum, millet, and the under-utilized grains that are less prone to mycotoxin contamination. 

## Figures and Tables

**Table 1:** Exposure assessment and estimated liver cancer risk due to consumption of maize grains.

AEZ	* Total Aflatoxins (ng/g)	^† ^PDI (ng·kg^−1^ bw·day^−1^)			^†† ^AFB_1_ Concentration	** Dietary Exposure to AFB_1_ (ng·kg^−1^ bw·day^−1^)			^‡‡^ MOE			^§§ ^Population Risk for Primary Liver Cancer (Cancer/Year/100,000 Population)		
		Infant	Children	^‡ ^Adult		Infant	Children	Adult	Infant	Children	Adult	Infant	Children	Adult
SS	137.3 ^§^	782.8	313.1	130.5	119.1	679.1	271.6	113.2	0.22	0.54	1.30	56.0	22.4	9.3
	530.0 ^¶^	3021.0	1208.4	503.5	489.8	2791.9	1116.8	465.3	0.06	0.14	0.34	230.3	92.1	38.4
NGS	196.4	1119.4	447.8	186.6	217.1	1237.2	494.9	206.2	0.15	0.38	0.91	102.1	40.8	17.0
	756.0	4309.2	1723.7	718.2	688.7	3925.3	1570.1	654.2	0.04	0.10	0.24	323.8	129.5	54.0
SGS	447.8	2552.7	1021.1	425.5	506.7	2888.3	1155.3	481.4	0.07	0.17	0.40	238.3	95.3	39.7
	2170.0	12,368.0	4947.6	2061.5	1967.7	11,216.1	4486.4	1869.3	0.01	0.03	0.08	925.3	370.1	154.2
DS	596.9	3402.1	1360.8	567.0	510.5	2909.6	1163.9	484.9	0.05	0.12	0.30	240.0	96.0	40.0
	7380.0	42,066.0	16,826.4	7011.0	6738.0	38,406.6	15,362.6	6401.1	0.00	0.01	0.02	3168.5	1267.4	528.1
HF	296.2	1688.5	675.4	281.4	270.1	1539.6	615.8	256.6	0.10	0.25	0.60	127.0	50.8	21.2
	569.0	3243.3	1297.3	540.6	516.4	2943.4	1177.4	490.6	0.05	0.13	0.31	242.8	97.1	40.5
National	334.9	1909.1	763.6	318.2	324.7	1850.7	740.3	308.5	0.12	0.3	0.70	152.7	61.1	25.5
	2281.0	13,001.5	5200.7	2167.0	2080.1	11,856.7	4742.7	1976.1	0.03	0.08	0.20	978.2	391.3	163.0

Values with different superscripts along the same column are significantly different at *p* < 0.05. * Summation of mean of means of aflatoxin B_1_, B_2_, G_1_ and G_2_ concentrations as reported by Adetunji et al. [3]. ^†^ Probable Daily Intake (PDI; Assumed 10 kg, 25 kg and 60 kg body weight for infants, children and adults, respectively) calculated by multiplying the aflatoxin concentration in maize grains by consumption rates of maize in Nigeria (57 g/person/day) as estimated by the World Health Organization [21] and divided by body weight of consumers. ^‡^ Adult PDI as reported by Adetunji et al. [9]. ^§^ Mean of means of aflatoxin concentration/AEZ. ^¶^ Mean of means of maximum concentration of aflatoxins/agro-ecological zone (AEZ: SS, Sudan Savanna; NGS, Northern Guinea Savanna; SGS, Southern Guinea Savanna; DS, Derived Savanna; HF, Humid Forest). ** Dietary exposure to aflatoxin B_1_ (AFB_1_), calculated by multiplying AFB_1_ concentration in maize with the consumption rate of maize and divided by the body weight of each population. ^††^ AFB_1_ concentration as reported by Adetunji et al. [3]. ^‡‡^ Margin of Exposure (MOE); calculated by dividing the benchmark dose lower limit (BMDL) for aflatoxins by AFB_1_ exposure. ^§§^ Population risk for primary liver cancer = Exposure ×Average potency (0.0825).

**Table 2:** Risk characterization of non-genotoxic and non-carcinogenic mycotoxins based on probable daily intake.

		Mycotoxin (×10^−3^ μg·kg^−1^ bw·day^−1^)											
AEZ	Daily Intake	Total Fumonisins (ΣFB_1_, FB_2_ and FB_3_) * (2 μg·kg^−1^ bw·day^−1^)			OchratoxinA * (17 ng·kg^−1^ bw·day^−1^)			Deoxynivalenol * (1 μg·kg^−1^ bw·day^−1^)			Zearalenone * (0.25 μg·kg^−1^ bw·day^−1^)		
		Infant	Children	Adult ^§^	Infant	Children	Adult ^§^	Infant	Children	Adult ^§^	Infant	Children	Adult ^§^
SS	^†^ PDI	3957.2	1582.9	659.5	119.9	48.0	20.0	311.3	124.5	51.9	0.0	0.0	0.0
	^‡^ %TDI	197.9	79.2	33.0	705.1	282.1	117.5	31.1	12.5	5.2	0.0	0.0	0.0
NGS	PDI	16,983.2	6793.3	2830.5	0.0	0.0	0.0	629.4	251.8	104.9	5.1	2.0	0.9
	%TDI	849.2	339.7	141.5	0.0	0.0	0.0	63.9	25.2	10.5	2.1	0.8	0.3
SGS	PDI	15,247.6	6099.0	2541.3	199.5	79.8	33.3	294.2	117.7	49.0	29.9	12.0	5.0
	%TDI	762.4	305.0	127.1	1173.5	469.4	195.6	29.4	11.8	5.0	12.0	4.8	2.0
DS	PDI	12,276.2	4910.5	2046.0	732.1	292.8	122.0	292.0	116.8	48.7	989.1	395.6	164.8
	%TDI	613.8	245.5	102.3	4306.5	1722.6	717.8	29.2	11.7	4.9	395.6	158.3	66.0
HF	PDI	13,759.0	5503.6	2293.2	0.0	0.0	0.0	386.0	154.4	64.3	0.0	0.0	0.0
	%TDI	688.0	275.2	114.7	0.0	0.0	0.0	38.6	15.4	6.4	0.0	0.0	0.0
National	PDI	12,444.6	8296.4	3456.8	193.1	140.2	58.4	637.6	255.0	106.3	341.3	136.5	49.3
	TDI	622.2	414.8	172.8	2061.7	824.7	343.6	64.1	25.5	10.7	136.5	54.6	27.3

* Tolerable Daily Intake. ^†^ Probable Daily Intake. ^‡^ %Tolerable Daily Intake. ^§^ Adult PDI as reported by Adetunji et al. [9].
